# Lidocaine ameliorates chronic constriction injury-induced neuropathic pain through regulating M1/M2 microglia polarization

**DOI:** 10.1515/med-2022-0480

**Published:** 2022-05-13

**Authors:** Jiaqi Yuan, Yue Fei

**Affiliations:** Department of Anesthesiology, Sir Run Run Shaw Hospital, Hangzhou, Zhejiang, China; Department of Anesthesiology, Sir Run Run Shaw Hospital, 3 East Qingchun Road, Jianggan District, Hangzhou, Zhejiang, China

**Keywords:** lidocaine, M1/M2 microglia polarization, neuropathic pain

## Abstract

This study is intended to explore the mechanism that lidocaine ameliorates chronic constriction injury (CCI)-induced neuropathic pain (NP) related to the polarization of M1 and M2 microglia. CCI rats were established by surgery to induce NP. The mechanical withdrawal threshold (MWT) and thermal withdrawal latency (TWL) of rats were determined. Microglial line HAPI cells were polarized into M1 or M2 cells using lipopolysaccharide (LPS) or interleukin (IL)-4, respectively. Immunofluorescence staining was performed to determine the Iba1/CD86- and Iba1/CD206-positive cells. Markers of M1 and M2 microglia were assessed using flow cytometry. Real-time polymerase chain reaction and enzyme-linked immunosorbent assay were performed to detect the level of mRNA and inflammatory factors. Lidocaine ameliorates CCI-induced NP, evidenced by the markedly increased values of MWT and TWL in NP rats. Lidocaine inhibited M1 microglia polarization but promoted M2 microglia polarization in a rat model of CCI-induced NP. Besides, in the *in vitro* experiment, lidocaine regulated M1/M2 polarization in LPS- or IL-4-treated HAPI microglia. Lidocaine ameliorates CCI-induced NP by regulating M1/M2 microglia polarization. This study investigated the biological role of lidocaine in regulating NP in rats, which may be helpful for revealing the pathogenic mechanisms of NP and provide a potential therapeutic factor.

## 1 Introduction

Neuropathic pain (NP) is defined as pain arising from a lesion or disease affecting the somatosensory system at either peripheral or central level, and it is a chronic condition. NP is manifested as spontaneous pain, hyperalgesia, allodynia, and secondary hyperalgesia [1]. It has been found that activated microglia in the spinal cord are involved in the development of NP by enhancing the protrusive transmission of neurons through a variety of pro-inflammatory factors and cell surface receptors [2]. Various components, such as neuregulin-1 [3], matrix metalloproteinase [4], and glial cell-derived neurotrophic factor [5], can activate microglia. The mechanism of microglia affecting NP has always been one of the focuses of medical science.

Microglia are intrinsic immune component cells of the central nervous system and belong to the mononuclear phagocyte system, which influence the neural environment maintenance, the development of the brain, and response to neural injury and repair [6]. For example, a previous study has shown that microglia exacerbate NP through TREM2/DAP12 signaling [7]. Other studies have indicated that neurons interact with microglia in NP through purinergic signaling [8] and that NP is affected by the crosstalk between microglia and astrocytes [9]. Importantly, a report suggested that estrogen reduces NP by inhibiting the activation of astrocytes and microglia [10]. Although signaling pathways and cytokines have been proved to provide various explanations for microglia activation, the precise mechanisms of microglia in NP are not fully revealed.

Under normal physiological conditions, microglia is in a quiescent state. When the body is subjected to external stimuli such as ischemia, nerve damage, or infection, resting microglia can sense the damage signal effectively, proliferate, migrate to the damaged area, and activate quickly. Under different microenvironments, activated microglia can appear as two polarized types with contrasting phenotypes and functions, namely the “classically activated” pro-inflammatory M1 type and the “alternative activated” anti-inflammatory M2 type [11]. Increasingly, studies have reported that inhibiting microglia polarization toward M1 and promoting it toward M2 can improve NP [12,13]. Therefore, regulation of the M1/M2 polarization direction of microglia is crucial for the progression and development of NP.

Lidocaine is a local anesthetic, which is often used in clinical practice because of its antiarrhythmic and nerve block effects. It was reported that lidocaine may attenuate inflammation in microglia and suppress NP [14,15]. Our previous study found that lidocaine ameliorated chronic constriction injury (CCI)-induced NP by activating astrocyte autophagy [16]. However, whether glial polarization is involved in the mechanism of lidocaine in the treatment of NP and neuroinflammation is unclear. Therefore, our study aims to investigate the mechanism by which lidocaine ameliorates CCI-induced NP related to the polarization of microglial M1 and M2. A rat model of CCI-induced NP was established and injected with lidocaine to observe its effect on microglia polarization in rats, and M1 or M2 microglia cells were, respectively, activated by lipopolysaccharide (LPS) or recombinant interleukin (IL)-4 and then treated with lidocaine to validate its effect on microglia polarization in the *in vitro* experiments. By providing the possible mechanism of lidocaine in the treatment of NP, this study may provide a new treatment strategy to reduce the pain of patients with NP.

## 2 Materials and methods

### 2.1 Animals and NP model

A total of 30 adult Sprague-Dawley (SD) rats (male: weighing 250–280 g, 8–10 weeks old) were purchased and kept in a standard SPF laboratory room (23 ± 1°C; 12 h light/12 h dark cycle; 45–55% humidity; and free access to food and water) for 2 weeks. SD rats were subjected to CCI surgery to induce NP, as reported previously [16]. In brief, SD rats were anesthetized (isoflurane), and then, the rats received blunt dissection surgery to expose the left (ipsilateral) common sciatic nerve at the midthigh level. After that, four silk threads (4–0) were loosely tied around the sciatic nerve (with intervals of ∼1 mm), and the incision area was closed. The left sciatic nerve of Sham-operated rats was exposed without silk ligation before tissue sampling. All protocols were approved by the Ethics Committee of Sir Run Run Shaw Hospital (SRRSH20220223).

### 2.2 Lidocaine administration and groups

On the third day postoperatively, intrathecal catheter implantation was performed as reported [14]. SD rats were randomly grouped: NP + Lidocaine group – rats received CCI surgery and lidocaine (50 μL; Sigma, St. Louis, MO, USA) at a concentration of 1.0% (dissolved in 0.9% saline); NP group – rats received CCI surgery and 50 μL of saline (0.9%); and Sham group – sham-operated rats received 50 μL of saline (0.9%). On the sixth day postoperatively, all the rats received lidocaine solution or saline (1 min; via the implanted catheter).

### 2.3 Test of mechanical withdrawal threshold (MWT) and thermal withdrawal latency (TWL)

The MWT and TWL of rats were tested using a von Frey electrical filament (IITC Life Science Inc, Woodland Hill, CA, USA) and Hot Sting instrument (Beijing, China), based on our previous study [14]. The mean MWT and TWL were from three readings calculated at each time point. All experimental rats were tested for MWT at 3, 7, 11, and 14 days postoperatively.

### 2.4 Microglia isolation

After the MWT and TWL tests, the spinal cord (L4–L6) was separated, and half of the tissues were used for immunofluorescence assay, and another half were used for isolation of microglia, as previously reported [12]. Briefly, the spinal cord (L4–L6) (∼1 mm^3^) was removed and chopped into small pieces (at room temperature). The tissues were incubated (0.1% trypsin; Gibco, Grand Island, NY, USA; 20 min; 37°C). The primary rat microglia suspensions were cultured (75 cm^2^ tissue culture flasks) and then pre-coated (5 mg/mL poly-d-lysine; Beyotime, Shanghai, China) in DMEM/F12 containing fetal bovine serum (FBS) (15%; Cayman Chemical Company, Ann Arbor, Michigan, USA), penicillin/streptomycin (1%; Gibco), and glutamine (1%; Gibco; 37°C). After 3 days of incubation, the culture medium was changed to DMEM/F12 containing FBS (10%) and incubated (48 h; 37°C).

### 2.5 Immunofluorescence assay

Rat spinal cord (L4–L6) was fixed (4% paraformaldehyde) and embedded (paraffin). After deparaffinization and rehydration processes, sections (5 μm) were obtained, placed (laser confocal small dish), and fixed (ice methanol; 15 min). After blocking, the primary antibodies, anti-Iba1 antibody (1:100, Abcam, Cambridge, MA, USA), anti-CD68 antibody (1: 100, Abcam), anti-CD86 antibody (1: 100, Abcam), anti-CD206 antibody (1:100, R&D Systems, Inc., Minneapolis, MN, USA), were added and incubated (overnight; 4°C). Then, the sections were washed with phosphate-buffered saline (PBS) several times and incubated with the secondary antibody (1 h) counterstained with 4,6-diamidino-2-phenylin-dole (DAPI) (1:1,000; Invitrogen; 5 min; room temperature) before being mounted. PBS was rinsed again, and the images were obtained (a confocal laser scanning microscope; Olympus, Tokyo, Japan). Fluorescent intensity was finally calculated.

### 2.6 Flow cytometry

Microglia cells were seeded in triplicate onto 6-well (1 × 10^5^ cells/well). Cells were blocked using the Ultra V blocker (Thermo Fisher Scientific, San Jose, CA, USA) and incubated with FITC anti-CD68, APC anti-CD86, or PE anti-CD206 antibodies (1:50). Finally, the cells were fixed and measured using a Guava easyCyte 8 Millipore flow cytometer.

### 2.7 Microglial line HAPI culture and treatment

Microglial line HAPI cells were purchased from Cobioer BioSciences Co., Ltd (Nanjing, China). Cells were thawed and passaged until logarithmic growth was achieved. Cell cultures were maintained (25–75 cm^2^ tissue culture flasks) (Corning-Costar Corp., Cambridge, Massachusetts, USA; 5–9 × 10^5^ cells/mL) in Dulbecco’s modified Eagle’s medium (DMEM) supplemented with FBS (10%), heat-inactivated (56°C; 30 min), l-glutamine (2 mM; Sigma), and 1× Pen–Strep (Sigma). The cells were passaged (every 3–4 days; 1 × 10^5^ cells/mL). The media were removed, and fresh FBS (10%)-supplemented DMEM was added to the cells.

The cells were seeded (24-well plates 5; ×10^4^ cells/well) and were divided into five groups. LPS group: for the generation of M1-polarized HAPI cells, cells were treated with LPS (100 ng/mL) for 24 h. IL-4 group: for the generation of M2-polarized HAPI cells, cells were treated with IL-4(20 ng/mL) for 24 h. LPS + Lidocaine group: HAPI cells were treated with LPS (100 ng/mL) combined with lidocaine (10 μg/mL) for 24 h. IL-4 + Lidocaine group: HAPI cells were treated with IL-4 (20 ng/mL) combined with lidocaine (10 μg/mL) for 24 h. Control group: normal HAPI cells treated with equivalent PBS for 24 h were served as Control. The plates were incubated (37°C; 5% CO_2_/95% air), and aliquots of supernatants were used for enzyme-linked immunosorbent assay (ELISA).

### 2.8 ELISA

The levels of tumor necrosis factor-α (TNF-α), IL-6, and IL-10 in cell supernatant were assessed using Rat TNF-α ELISA Kit, Rat IL-6 ELISA Kit, or Rat IL-10 ELISA Kit (Elabscience, Wuhan, China) as per the manufacturers’ instruction. The optical density values of the samples were measured using an enzyme marker (Thermo Fisher Scientific, San Jose, CA, USA).

### 2.9 Real-time PCR

The total RNA was extracted using TRIzol (Invitrogen, Carlsbad, CA, USA) and converted into first-strand cDNA using PrimeScript^TM^ RT reagent Kit (TaKaRa Biotechnology, Co., Ltd., Dalian, China). Quantitative real-time polymerase chain reaction (qRT-PCR) experiments were performed with SYBR Green I in a Light Cycler 96 (Roche Applied Science, Mannheim, Germany). The primers were as follows: iNOS, 5′-CCCTTCCGAAGTTTCTGGCAGCAGC-3′ and 5′-GGCTGTCAGAGCCTCGTGGCTTTGG-3′; Arg-1, 5′-CCAGAAGAATGG AAGAGTCAGTGT-3′ and 5′-GCAGATATGCAGGGAGTCACC-3′; and GAPDH, 5′-GCACCACCAACTGCTTAGCA-3′ and 5′-GTCTTCTGGGTGGCAGTGATG-3′. For normalization, the GAPDH gene was used as a reference gene and calculated using the 2^−ΔΔCt^ method.

### 2.10 Statistical analysis

All results were shown as mean ± SD and analyzed using statistical product and service solutions (SPSS, version 20.0) and GraphPad Prism 7. All experiments were performed three times. The comparisons between two groups or among multi-groups were analyzed using a two-tailed Student’s *t*-test or a two-way ANOVA followed by Bonferroni’s *post hoc* test; *p* < 0.05 was considered statistically significant.

## 3 Results

### 3.1 Lidocaine ameliorates CCI-induced NP

**Figure 1:**
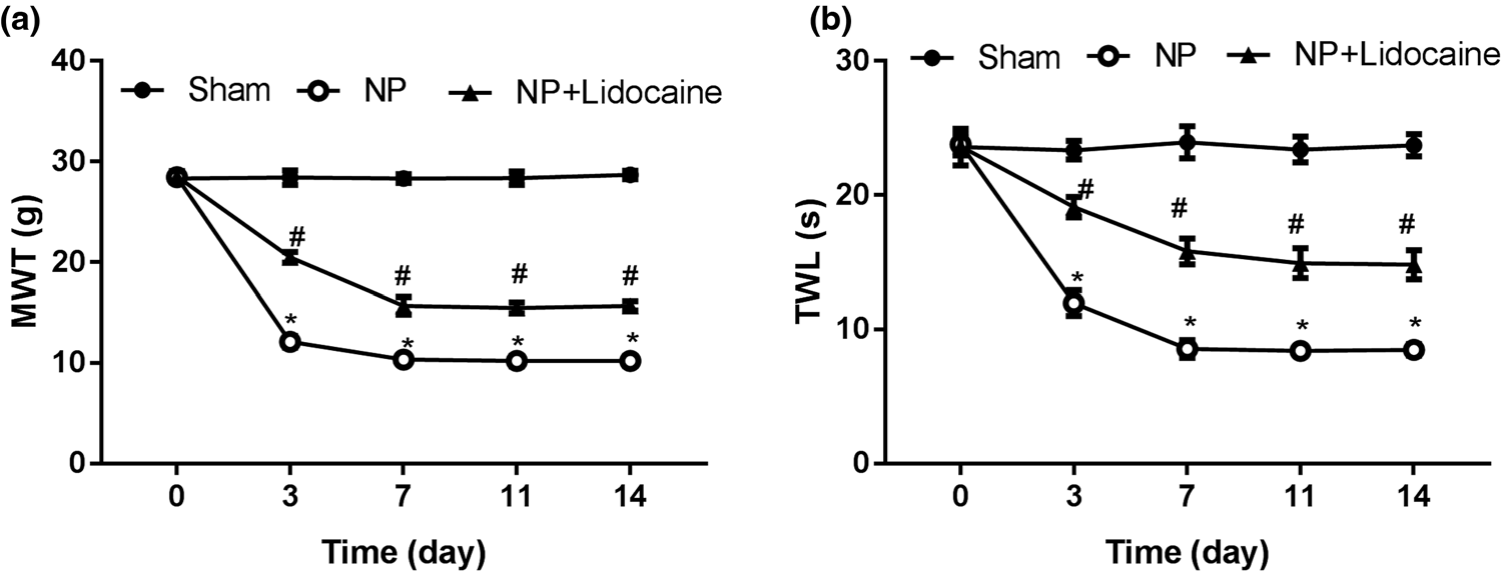
Lidocaine ameliorates CCI-induced NP: (a) The value of MWT was detected in NP rats at 3, 7, 11, and 14 days. (b) The value of TWL was detected in NP rats at 3, 7, 11, and 14 days after induction of CCI. *N* = 10. Data are expressed as mean ± SD and were analyzed using one-way ANOVA with Tukey’s *post hoc* test. *p < 0.05 versus Sham group; #*p* < 0.05 versus NP group.

As shown in Figure 1, the values of MWT and TWL at 3, 7, 11, and 14 days were, respectively, lower in the NP group than that in the Sham group. However, after treatment with lidocaine, the values of MWT and TWL were markedly increased.

### 3.2 Lidocaine regulated M1/M2 microglial polarization in a rat model of CCI-induced NP

**Figure 2:**
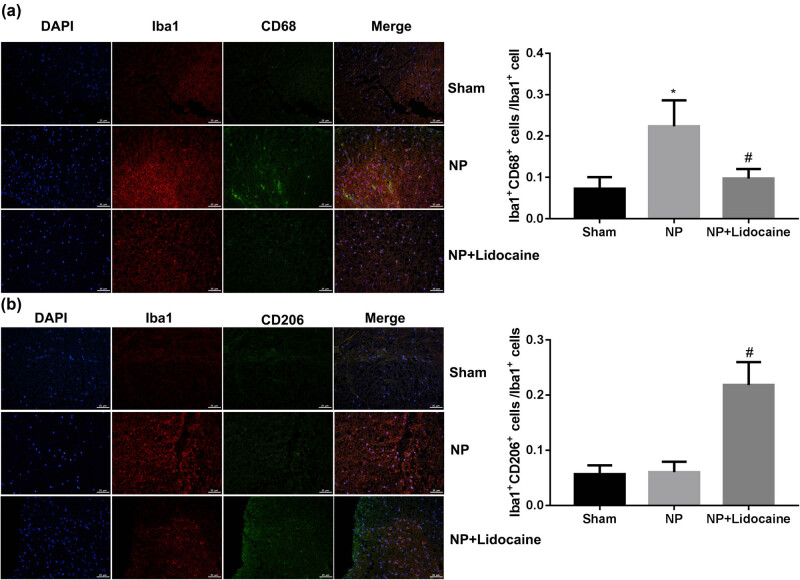
Lidocaine regulated M1/M2 microglial polarization in a rat model of CCI-induced NP. The rats in different groups were sacrificed at 14 days, and L4–L6 segments of the rat spinal cord were separated. Representative images of immunofluorescence assay were used to detect the microglia marker Iba1, M1 marker CD68, and M2 marker CD206 to quantify the number of M1 (a) and M2 microglia (b). DAPI (nuclei, blue), total microglia (Iba-1, red), M1-microglia phenotype (CD68, green), and M2-microglia phenotype (CD206, green). Data are expressed as mean ± SD and were analyzed using one-way ANOVA with Tukey’s post hoc test. *p < 0.05 versus Sham group; #*p* < 0.05 versus NP group.

The rats in different groups were sacrificed at 14 days, and L4–L6 segments of rat spinal cord were separated. Immunofluorescence assay was used to detect the microglial marker Iba1, M1 marker CD68, and M2 marker CD206 to quantify the number of M1 and M2 microglia. Figure 2 shows that the proportion of lba1^+^ CD68^+^/lba1^+^ was significantly increased in the NP group, while was decreased with lidocaine treatment. On the contrary, the proportion of Iba1^+^ CD206^+^/Iba1^+^ showed no significant differences between Sham and NP groups, while was increased with lidocaine treatment.

**Figure 3:**
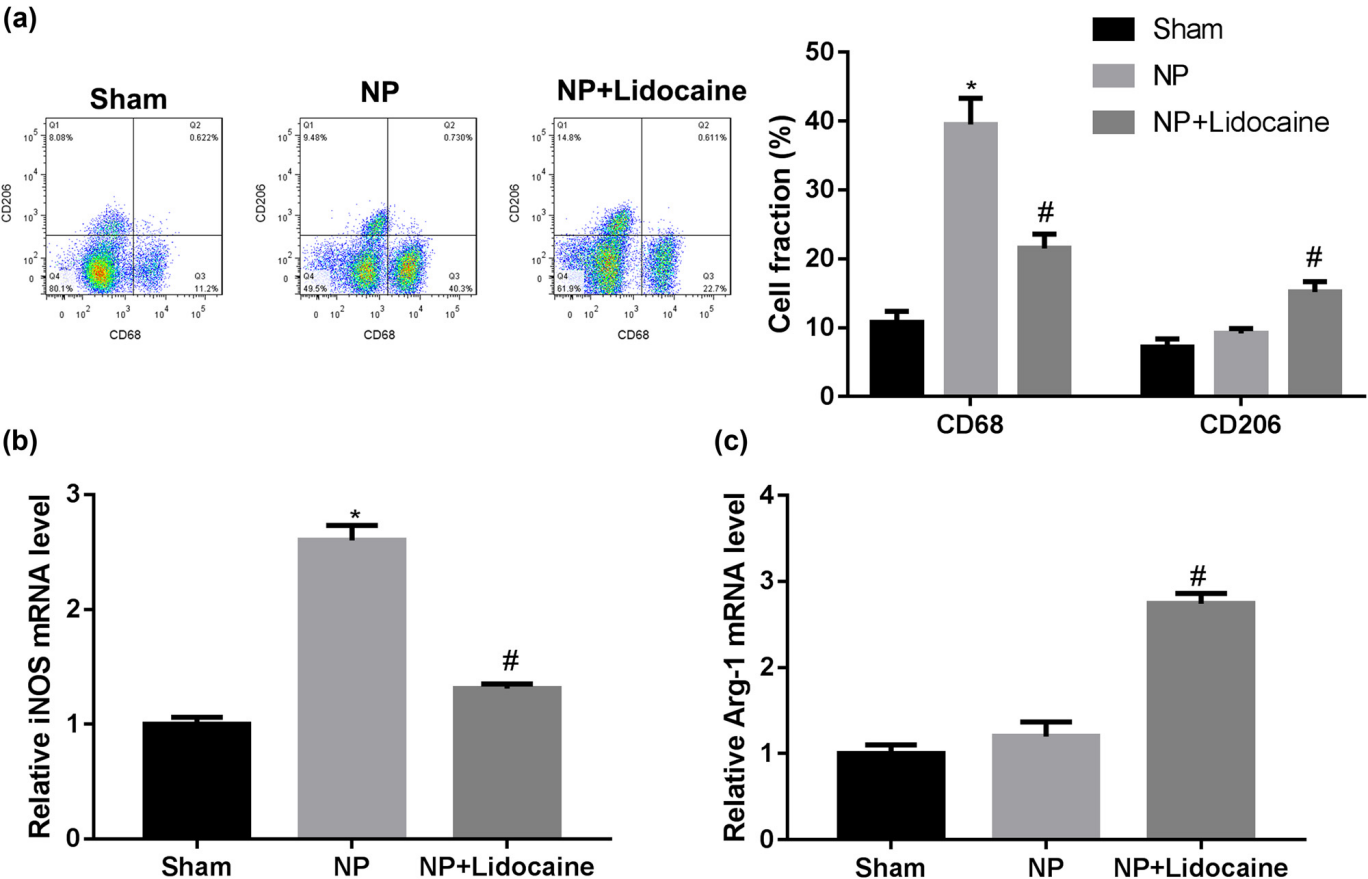
Lidocaine regulated M1/M2 microglia polarization in a rat model of CCI-induced NP. The rats in different groups were sacrificed at 14 days, and L4–L6 segments of the rat spinal cord were separated and microglia were isolated. (a) Flow cytometry was used to detect the proportion of M1 and M2 microglia, as evidenced by the fraction of CD68^+^ cells and CD206^+^ cells. RT-PCR was used to detect the expression of M1 marker iNOS (b) and M2 marker Arg-1 (c). Data are expressed as mean ± SD and were analyzed using one-way ANOVA with Tukey’s *post hoc* test.*p < 0.05 versus Sham group; #*p* < 0.05 versus NP group.

Then, the microglia were isolated from the spinal cord, and the flow cytometry was used to detect the proportion of M1 and M2 microglia. Consistently, lidocaine treatment decreased the fraction of CD68^+^ microglia but increased the fraction of CD206^+^ microglia (Figure 3a). RT-PCR was used to detect the expression of M1 marker iNOS and M2 marker Arg-1. As shown in Figure 3b and c, lidocaine treatment decreased the mRNA expression level of iNOS but increased the mRNA expression level of Arg-1.

### 3.3 Lidocaine regulated M1/M2 polarization in microglia cell

**Figure 4:**
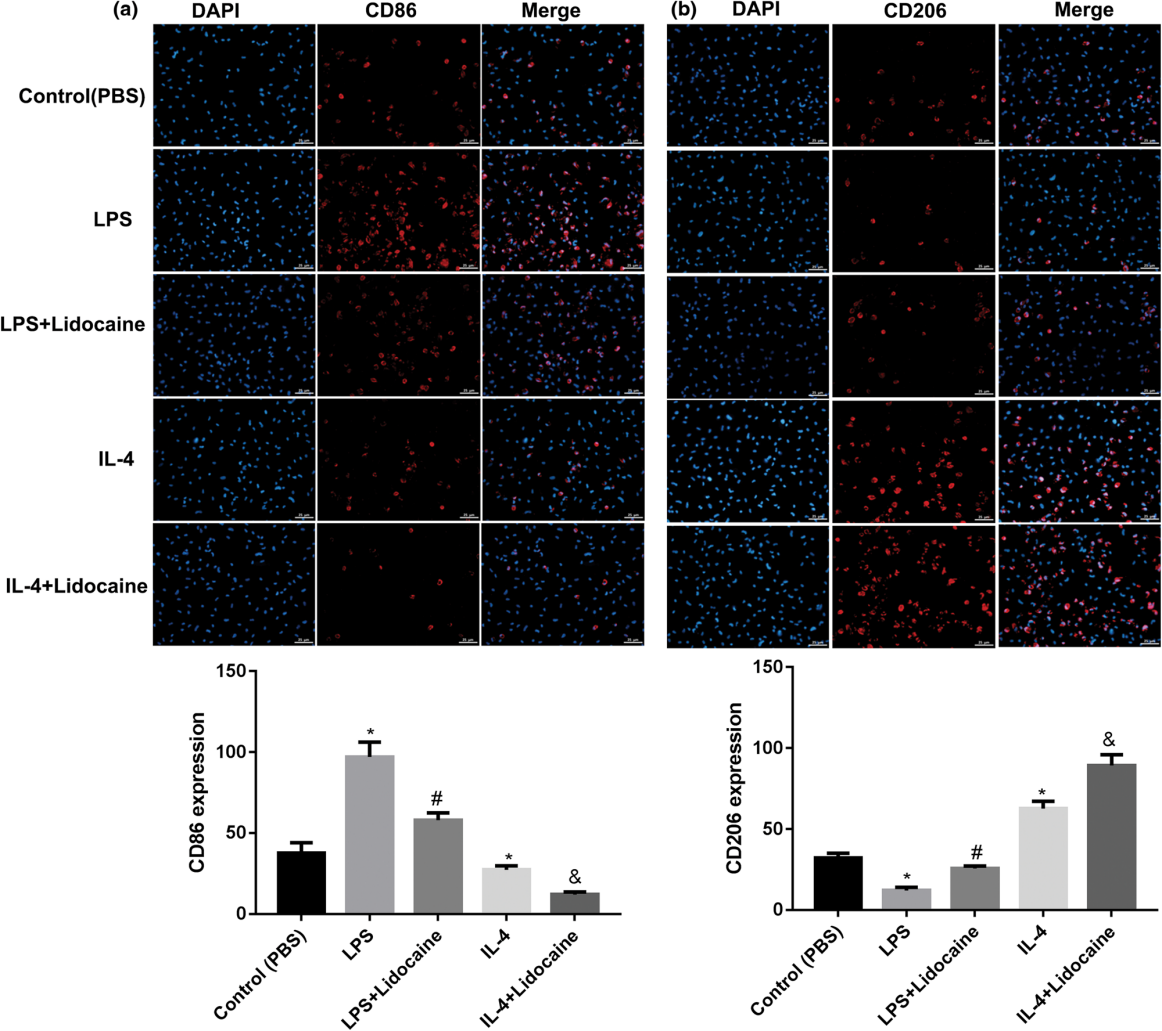
Lidocaine regulates M1/M2 microglia polarization *in vitro.* The expression levels of M1 and M2 cell markers (a) CD86 and (b) CD206, in LPS- or IL-4-treated HAPI microglia were detected using immunofluorescence and qualified. Data are expressed as mean ± SD and were analyzed using one-way ANOVA with Tukey’s *post hoc* test. *p < 0.05 versus Control group; #*p* < 0.05 versus LPS group; and &*p* < 0.05 versus IL-4 group, three independent experiments.

To validate the effect of lidocaine on microglia polarization *in vitro*, the HAPI cells were stimulated by LPS to an M1 phenotype or by IL-4 to an M2 phenotype, and then treated with lidocaine. The expression levels of M1 and M2 cell markers, CD86 and CD206, were detected using immunofluorescence. As shown in Figure 4a, the number of CD86 was significantly increased in the LPS group, while was decreased with lidocaine treatment. On the contrary, the number of CD86 was significantly decreased in the IL-4 group and further decreased by lidocaine treatment. Besides, the number of CD206 was significantly decreased in the LPS group, while was increased by lidocaine treatment. However, the number of CD206 was significantly increased in the IL-4 group and further increased by lidocaine treatment (Figure 4b). The results suggested that lidocaine could inhibit microglial M1 polarization and promote microglial M2 polarization.

**Figure 5:**
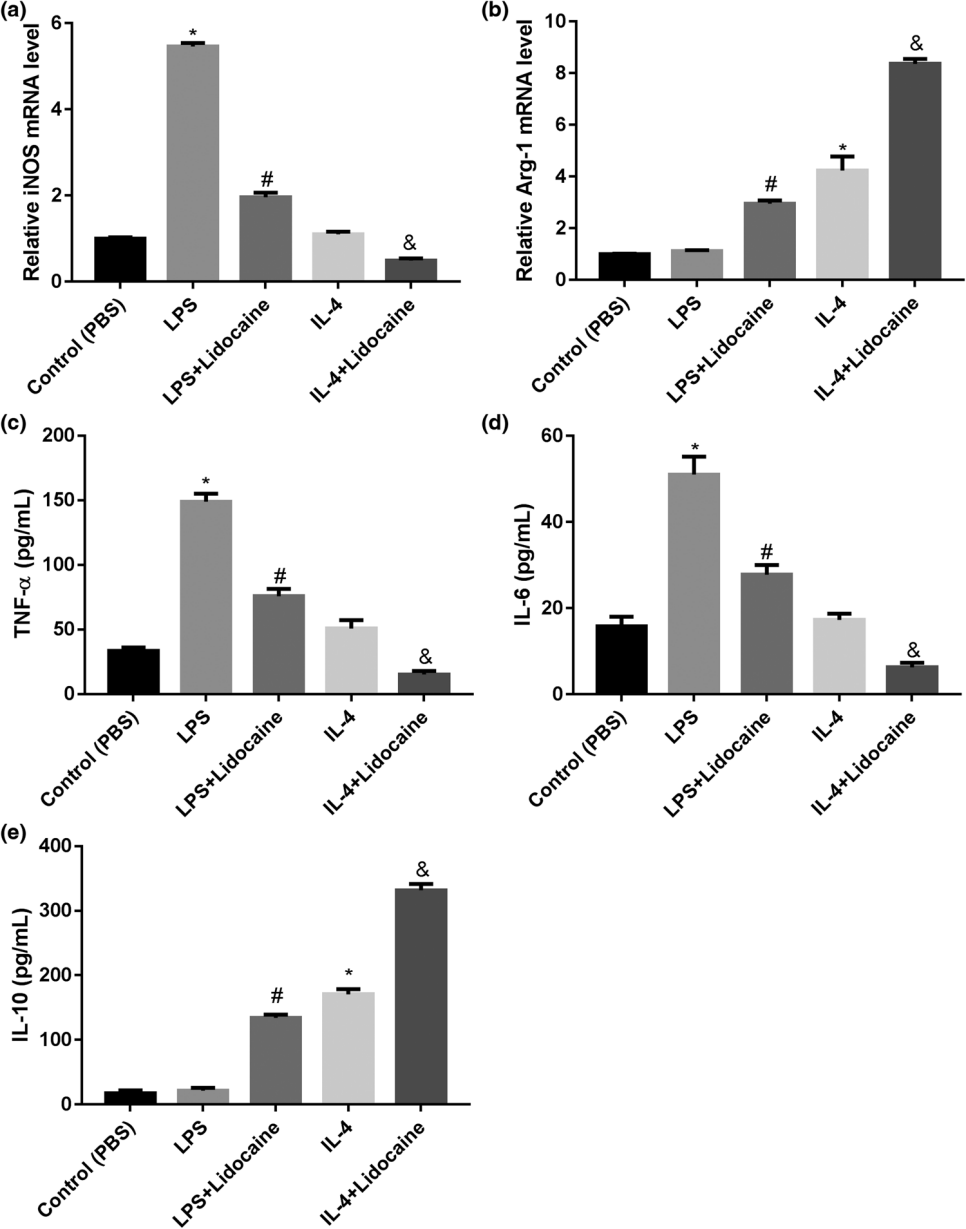
Lidocaine inhibits M1 polarization but promoted M2 polarization in HAPI microglia. The expression levels of (a) M1 marker iNOS and (b) M2 marker Arg-1 in LPS- or IL-4-treated HAPI microglia were measured using qRT-PCR. ELISA was used to detect (c) TNF-α, (d) IL-6, and (e) IL-10 levels in the culture supernatant of LPS- or IL-4-treated HAPI microglia. Data are expressed as mean ± SD and were analyzed using one-way ANOVA with Tukey’s *post hoc* test. *p < 0.05 versus Control group; #*p* < 0.05 versus LPS group; and &*p* < 0.05 versus IL-4 group, three independent experiments.

The expression levels of M1 marker iNOS and M2 marker Arg-1 were measured using qRT-PCR. ELISA was used to detect TNF-α, IL-6, and IL-10 levels in the culture supernatant. Accordingly, LPS treatment significantly increased the mRNA expression of iNOS and the levels of TNF-α and IL-6, and IL-4 treatment significantly increased the mRNA expression of Arg-1 and induced the level of IL-10. However, lidocaine treatment reversed the effect of LPS, but enhanced the effect of IL-4 (Figure 5). These data further indicated that lidocaine inhibited microglial M1 polarization and promoted microglial M2 polarization.

## 4 Discussion

Here, the study showed that the MWT and TWL values in NP rats were lower than those in Sham rats, whereas lidocaine administration increased these values, which directly confirmed the alleviative effect of lidocaine on the progression and development of NP. The analgesic effect of lidocaine on NP was consistent with previous studies [17,18,19].

Activated microglia in the spinal cord after nerve injury can enhance synaptic transmission of spinal cord dorsal horn neurons through various cell surface receptors and pro-inflammatory factors, thus participating in the onset and development of NP [20]. Here, a significant increase in Iba-1+ cells (microglial markers) in the L4–L6 spinal cord segments of rats after CCI was observed, indicating that microglia are activated after NP. Our results are consistent with previous studies [21,22]. However, lidocaine significantly decreased the number of Iba-1^+^ cells, indicating its role in suppressing microglial activation.

Besides, the number of Iba1^+^ CD68^+^ was significantly increased in the NP group, while was decreased with lidocaine treatment. On the contrary, the number of Iba1^+^ CD206^+^ showed no significant differences between Sham and NP groups, while was increased with lidocaine treatment. The flow cytometry and determination of the levels of M1 and M2 markers showed consistent results. Microglia can be activated into M1 or M2 type by LPS or Il-4 stimulation, which secretes pro-inflammatory cytokines or is responsible for the resolution of inflammation and tissue repair, respectively [23,24]. *In vitro* experiments of this study confirmed that lidocaine suppressed microglial M1 polarization, while promoted microglial M2 polarization. Determination of the levels of M1 (iNOS, IL-1β, and TNF-α) and M2 markers (Arg-1 and IL-10) showed consistent results. It is well known that factors secreted by activated microglia affect the M1 polarization states by autocrine (IL-6, IL-1β, and TNF-α), which cause the painful symptoms; IL-10 released by activated microglia causes M2 polarization [25]. These results suggested that lidocaine decreased microglial M1 polarization and increased microglial M2 polarization, thereby alleviating microglia activation and exerting protective effects on NP.

Compelling studies have suggested that switching the polarization of microglia from a pro-inflammatory M1 phenotype to an anti-inflammatory M2 phenotype is a novel strategy for NP treatment [26,27,28,29]. Besides, Willemen et al. [29] suggested that an increased ratio of M1/M2-type markers in spinal microglia/macrophages was related to the persistent hyperalgesia in G protein-coupled receptor kinase (GRK2)-deficient mice. However, intraperitoneal miR-124 treatment alleviated NP by restoring spinal M1/M2 markers. Wang et al. [30] reported that dual specificity phosphatase 1 (DUSP1) switched microglial M1 to M2 polarization in the medial prefrontal cortex and attenuated CCI-induced NP by inhibiting the MAPK signaling. Gui et al. [31] proved that BTX-A promotes microglial M2 polarization and suppresses CCI-induced NP by inhibiting the P2X7 receptor. Zhang et al. [32] indicated that suppression of miR-155 attenuates NP by inducing an M1 to M2 switch in microglia. Only a limited number of mechanisms have been revealed in the article, and there are still many potential mechanisms to be further validated, such as Akt signaling [33], sphingosine 1-phosphate/S1P receptors [34], and T-cell immunoglobulin mucin 3/galectin-9 [35].

## 5 Conclusion

To our knowledge, this study demonstrates for the first time that lidocaine administration regulates M1/M2 microglial polarization, thereby alleviating microglia activation and exerting protective effects on NP. This study investigated the biological role of lidocaine in regulating NP in rats, which may help reveal the pathogenic mechanisms of NP and provide a potential therapeutic factor.

## Supplementary Material

Supplementary Table

Supplementary Figure
