# Supplementary figures and images for: Lidocaine ameliorates chronic constriction injury-induced neuropathic pain through regulating M1/M2 microglia polarization
Source: Open Med (Wars). 2022 May 13;17(1):897–906. doi: 10.1515/med-2022-0480 (PMC9106111; doi:10.1515/med-2022-0480)

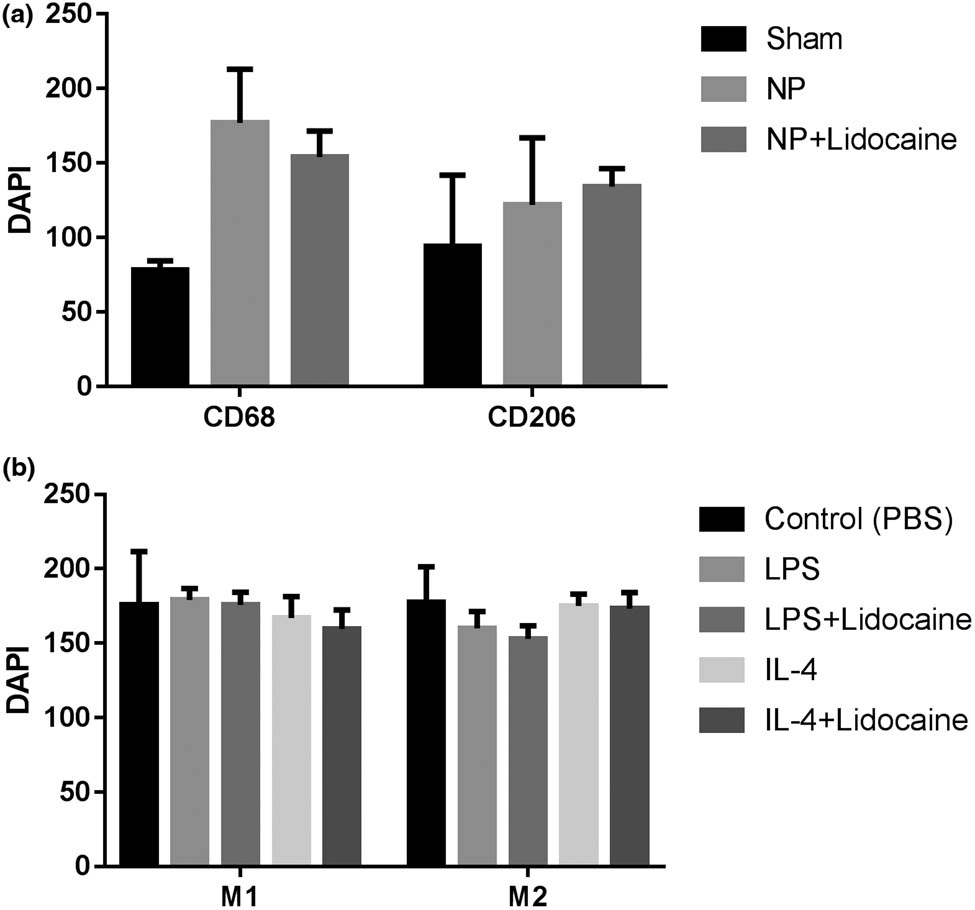

Supplement: Supplementary Figure [file med-2022-0480-sf1.jpg]
